# Multiplex Analysis of Pro- or Anti-Inflammatory Serum Cytokines and Chemokines in relation to Gender and Age among Tanzanian Tuberculous Lymphadenitis Patients

**DOI:** 10.1155/2015/561490

**Published:** 2015-04-28

**Authors:** Tehmina Mustafa, Karl Albert Brokstad, Sayoki G. Mfinanga, Harald G. Wiker

**Affiliations:** ^1^Centre for International Health, Department of Global Public Health and Primary Care, University of Bergen, 5021 Bergen, Norway; ^2^Department of Thoracic Medicine, Haukeland University Hospital, 5021 Bergen, Norway; ^3^Broegelmann Research Laboratory, Department of Clinical Science, University of Bergen, 5021 Bergen, Norway; ^4^National Institute for Medical Research, Muhimbili, Tanzania; ^5^The Gade Research Group for Infection and Immunity, Department of Clinical Science, University of Bergen, 5021 Bergen, Norway

## Abstract

*Objectives*. Tuberculous lymphadenitis is the most common form of extrapulmonary tuberculosis (TB) with a female and paediatric preponderance, postulated to be due to differences in the immune response. The aim of this study was to analyze the differences in the serum cytokine levels of tuberculous lymphadenitis patients with respect to age and gender. *Methods*. A multiplex bead-based enzyme-linked immunosorbent assay was used to measure IFN-*γ*, TNF-*α*, GM-CSF, IL-1*β*, IL-2, IL-4, IL-5, IL-6, IL-8, IL-10, IL-12, IL-15, and IL-17 levels in sera of patients (*n* = 86) and healthy controls (*n* = 23). *Results*. Levels of IFN-*γ*, TNF-*α*, GM-CSF, IL-1*β*, IL-2, IL-4, and IL-6 were higher in adult patients than in controls, while those of IL-12 were lower (*P* < 0.05). Children had lower levels of TNF-*α*, GM-CSF, and IL-5 and higher levels of IL-2 compared with adult patients (*P* < 0.05). The male adult patients had higher levels of IL-17 and lower levels of IL-12 compared with female adult patients (*P* < 0.05).  *Conclusion*. There were significant differences in the levels of circulating cytokines with respect to gender and age. Children had generally lower levels of cytokines as compared to adults, which could make them more susceptible. Findings do not support that female preponderance is due to differences in immune response.

## 1. Introduction

Extrapulmonary tuberculosis (TB) constitutes about 15 to 20% of all cases of TB. The true rate may be even higher due to incomplete reporting in many developing countries. The annual global incidence of EPTB has been increasing in last decades due to the changing TB control practices, spread of Human Immune Deficiency Virus (HIV), and the population growth. Lymphadenitis is the most common form of extrapulmonary TB with a female and paediatric preponderance [1–4]. Children belong to the category of relatively susceptible individuals to develop TB. The mechanisms by which women and children become more susceptible to develop TB lymphadenitis are not fully understood. TB lymphadenitis is usually a self-contained disease and there is a granulomatous immune response in the lymph nodes which is considered to be a correlate of protective immunity. Formation of granulomas is achieved by cell-mediated immunity orchestrated by a complex interplay of cytokines and chemokines [5]. However, despite an effective cell-mediated immunity, eradication of the pathogen is not achieved and the disease runs a chronic course [6]. Mechanisms involved in the regulation of immune responses in TB lymphadenitis are not clear, and the knowledge of systemic levels of different cytokines is limited. Few studies have sought to determine the cytokine balance at the systemic level in a small number of patients with TB lymphadenitis by using the* ex vivo* cytokine production capacity of isolated peripheral blood mononuclear cells or CD4^+^ T cells after stimulation [7, 8]. The* ex vivo* stimulated production of cytokines does not necessarily provide insight into the actual status of the cytokine network* in vivo*. Indeed, in patients with severe bacterial infections much of the observed organ injury is considered to be related to enhanced* in vivo* production of proinflammatory cytokines, while peripheral blood mononuclear cells isolated from those patients produce significantly less cytokines upon stimulation as compared to cells from healthy controls [9]. We therefore decided to investigate unstimulated serum samples from TB lymphadenitis patients. The aim was to compare the immune response among TB lymphadenitis patients with respect to gender and age. A cytokine panel which best represented the spectrum of immune process involved in TB was analysed by multiplex bead-based enzyme-linked immunosorbent assay. This included the Th1 (INF-*γ*, TNF-*α*) versus Th2 (IL-4, IL-5) balance, T-cell stimulation (IL-2, IL-12), macrophage activation (IL-1*β*), granuloma formation (IL-8), limitation of inflammation (IL-10), and other inflammatory cytokines and chemokines (IL-6, GM-CSF, IL-15, and IL-17).

## 2. Material and Methods

### 2.1. Patients

This study was conducted using a serum bank from patients diagnosed with TB lymphadenitis. These patients were recruited in an epidemiological study from four districts: Babati, Karatu, Hanang, and Mbulu of the Karatu region, Tanzania [10]. These patients were small-scale farmers, cattle-keepers, and nomads. The characteristics of the study population are provided in Table 1.

Diagnosis of TB lymphadenitis was based on strong clinical evidence according to the National Tuberculosis and Leprosy Control Programme clinical guidelines [11], that is, history of TB exposure, history of chronic relapsing fever, weight loss, and cervical swelling not responding to the common antibiotics. This was followed by the decision by a clinician to treat with a full course of anti-TB chemotherapy. The majority of patients presented with swelling in the neck. Other symptoms such as fever, pain, and weight loss were infrequent. About 50% of patients gave a history of TB in the family. History of previous TB treatment was given by about 1% of patients. Cervical lymph nodes were the main lymph nodes affected, enlarged in about 89% of the cases, while the axillary, inguinal, and mesenteric lymph nodes were involved in a small proportion of cases [10]. In only 1% of patients there was concomitant pulmonary TB, and in 99% of patients no pulmonary or another extrapulmonary spread than lymphadenitis was observed.

Sera from 23 healthy Tanzanian blood donors aged between 18 and 70 were used as controls. These sera were obtained from the blood bank at Muhimbili Medical Centre, Dar es Salaam, Tanzania [12]. Skin tests or IGRA test for mycobacterial infection was not performed in the study subjects. BCG vaccination status was determined by history of vaccination and the presence of vaccination scar.

### 2.2. Ethics Statement

Ethical clearance was obtained from the National Medical Research Coordinating Committee in Tanzania. The project was discussed with and was exempted from ethical clearance in Norway as the principal investigator and the patient material were from Tanzania. All the participants provided their consent to participate in this study. In case of minors, consent was obtained from the parents. The consent was verbal as the study participants were from rural Tanzania and could not read or write. This consent form was approved by the ethics committee. The normal control sera used in the study were obtained from the biobank left after the completion of another study on the blood donors from Tanzania [12]. These sera were stored at the University of Bergen and permission to use these in this study was obtained from the responsible persons in Tanzania and Norway.

### 2.3. Collection and Storage of Blood Serum

Blood was drawn from the patients using standardized phlebotomy procedures. Handling and processing were similar for all patients. Blood samples were collected without anticoagulant into 10 mL BD vacutainer Z (Becton Dickinson & Company, NJ, USA) and allowed to coagulate for 20 to 30 minutes at room temperature. The sera were separated and transferred to NUNC tubes (NUNC/Thermo Fischer, Roskilde, Denmark) and stored at −20°C. The NUNC tubes were labelled with identification numbers to ensure the confidentiality of the results. The samples were later shipped to the University of Bergen, Norway, where they were aliquoted and stored at −20°C.

### 2.4. Culture and HIV Test Procedures

Open biopsy specimens were taken from all patients before starting any anti-TB chemotherapy. The specimens were divided into two, one for culture and another for histology. The specimen for culture was placed in a universal container and stored in a deep freezer. These frozen biopsy specimens were processed for culture using aseptic techniques in a safety cabinet. All specimens were decontaminated, digested by standard procedures, and inoculated onto Lowenstein-Jensen egg medium under 37°C incubation for at least eight weeks.

For HIV testing, pre- and posttest counselling were conducted, and patients were assured that the results would be handled confidentially. HIV testing was performed using single Behringer ELISA tests (Dade, Behring Marburg GmbH, Emil-von-Behring Marburg, Germany). Positive cases were repeated with Wellcozyme HIV Recombinant (Murex Biotech, UK). Tests were conducted according to the manufacturer's protocol. Mycobacterial cultures were conducted at the Central Tuberculosis Reference Laboratory (CTRL) at NIMR Muhimbili Medical Centre, and HIV testing at National Reference Laboratory, at Muhimbili University of Health and Allied Sciences, Tanzania.

### 2.5. Multiplex Cytokine Bead-Based Enzyme-Linked Immunosorbent Assay (ELISA)

To detect cytokines in the sera, a human cytokine thirteen-plex antibody bead assay (Biosource, Camarillo, CA, USA) was used according to the manufacturer's instructions with a Luminex 100 System. IFN-*γ*, TNF-*α*, GM-CSF, IL-1*β*, IL-2, IL-4, IL-5, IL-6, IL-8, IL-10, IL-12, IL-15, and IL-17 were quantified (pg/mL). A standard curve was created from threefold dilution series of premixed standards. The assay was performed in a 96-well filter bottom plate supplied with the kit. Premixed beads coated with the target antibodies were added to each well, followed by the 50 *μ*L of incubation buffer. 100 *μ*L/well of the assay diluent was subsequently added. Premixed standards and samples were then added to the wells and incubated for 2 hours. Subsequently premixed biotinylated detector antibody was added to each well and incubated for 1 hour, followed by incubation with Streptavidin-RPE for 30 minutes. All steps were performed at room temperature, and the samples and reagents were kept in the dark during the procedure. Between each step the plates were washed twice and each incubation step was performed on a rotating platform (600 rpm).

### 2.6. Data Management and Statistical Analysis

Statistical analysis was conducted using SPSS for Windows. A nonparametric Mann-Whitney test was used for two group comparisons. Spearman's rank correlation was performed to determine the relationship between two variables. *P* values less than 0.05 were considered significant.

## 3. Results

### 3.1. Cytokines/Chemokines in Adult Patients

Table 2 shows the cytokine levels in the adult TB lymphadenitis patients and healthy blood donors from Tanzania. Serum levels of IFN-*γ*, TNF-*α*, GM-CSF, IL-1*β*, IL-2, IL-4, and IL-6 were higher in the patients compared with healthy controls, while IL-12 levels were lower. Serum levels of IL-5, IL-8, IL-10, IL-15, and IL-17 were not statistically different in the two groups. There was a large variation in the amount of different cytokines in the sera of both patients and controls.  Table 3 shows the correlations between various cytokines and chemokines. There was a positive correlation among IFN-*γ*, TNF-*α*, GM-CSF, IL-1*β*, IL-6, and IL-8. Th1 cytokines IFN-*γ* and TNF-*α* also correlated positively with the anti-inflammatory cytokine IL-10.

### 3.2. Differences in the Cytokine/Chemokines between Adults and Children

The levels of cytokines were generally lower in children as compared to adults with exception of IL-2 which was higher (Table 2). However, the statistically significant differences were only observed between serum levels of TNF-*α*, GM-CSF, and IL-5 and IL-2. There was a significant positive correlation among IFN-*γ*, TNF-*α*, GM-CSF, IL-6, and IL-8. Unlike the adult population, a negative correlation was observed between IL-5 and IL-12 and no significant positive correlation was observed between IFN-*γ* and TNF-*α* with anti-inflammatory IL-10 (Table 4).

### 3.3. Difference in Cytokines/Chemokines between Male and Female Adult Patients

The female adults had significantly lower levels of IL-17 and higher levels of IL-12 compared with the male adult patients (Table 5).

### 3.4. Relation of Cytokine/Chemokine Levels with Mycobacterial Culture

As detection of mycobacteria by culture reflects the lesions with higher bacillary load, a comparison was made between he culture-positive and culture-negative cases to see how this difference is reflected in the immune response. Among the adult patients, 13 (21%) cases were culture-positive and these cases had higher level of IL-12 compared with culture-negative cases (Table 5). IL-12 correlated positively only with IL-15 in culture-positive patients. The levels of the other cytokines were not statistically different. Among children culture results were available for 23 cases and 3 (13%) cases were culture-positive. The levels of cytokines were not statistically different between the two groups. When the adult and paediatric patients were combined, the difference in culture-positive and culture-negative cases was similar to that in the adult patients.

## 4. Discussion

Pathogenic mycobacteria are known to stimulate the immune response in such a way that the eradication of the pathogen is not fully achieved and the inappropriately simulated immune response leads to tissue destruction and the progression of disease rather than achieving the eradication of infection [5, 13–15]. In this study there were higher levels of the proinflammatory cytokines IFN-*γ*, IL-2, and TNF-*α*, and the chemokines GM-CSF, IL-1*β*, and IL-6 among TB cases as compared to controls. The levels of the anti-inflammatory cytokines IL-10 and IL-5 which are expected to balance the proinflammatory cytokines were not different between TB cases and controls. These findings imply that a relative lack of anti-inflammatory cytokines and thereby reduced inhibition of immune response to* M. tuberculosis* may be responsible for disease progression.

Lower levels of TNF-*α* and GM-CSF in sera from children compared with adults may indicate that the immune response in children may not be as effective as that in adults to control TB as both GM-CSF and TNF-*α* have been associated with increased resistance to mycobacterial infection [16, 17]. This may explain an increased preponderance of TB lymphadenitis among children. A recent study has indeed shown that healthy children generally secrete lower levels of cytokines as compared to adults [18]. One weakness of the study is that we do not have control sera from age-matched healthy children. Therefore, the serum levels of pediatric patients were not compared with the controls.

A higher female preponderance for TB lymphadenitis in adults has been suggested to be due to differences in the immune response [2, 19]. In this study the female patients had lower levels of IL-17 and higher levels of IL-12 compared with adult male patients. Production of IL-17 is shown to be negatively regulated by IL-12 in human T cells [20]. Recent findings suggest a role for IL-17-producing Th17 cells in TB with an early but transient Th17 burst which apparently contributes to protection whereas long lasting Th17 activity causes pathology [21–23]. The balance between IL17 and IL12 may partially explain the increased susceptibility of females as compared to males. However due to large dispersion in the data and several observations below the detection limit of the assay, it is difficult to make any firm conclusion. Further studies are required to understand the significance of these differences.

In this study, IL-12 p40 concentrations were lower in patients with active TB than in healthy controls. Paradoxically, patients with positive cultures had significantly higher IL-12 serum levels than culture-negative TB patients. Considering the essential role of IL-12 in a protective immune response to TB and that positive cultures are associated with a higher mycobacterial burden, it is difficult to explain this finding. IL-12 correlated positively only with IL-15 in all patients and in culture-positive patients. These two cytokines have been shown to activate natural killer cell function [24]. However no difference in the levels of other cytokines/chemokines between culture positive and negative patients suggests that TB lymphadenitis severity may result mainly from the immune response rather than the bacterial load in the tissues.

## 5. Conclusion

As compared with the controls, TB lymphadenitis patients secreted more proinflammatory cytokines and chemokines, except IL-12, while the levels of the anti-inflammatory cytokines were not different suggesting the role of inappropriate immune stimulation in the disease pathogenesis. There were significant differences in the levels of circulating cytokines with respect to gender and age. Children had generally lower levels of cytokines as compared to adults, and significantly lower levels of TNF-*α* and GM-CSF indicate that the immune response in children may not be as effective as that in adults which could make them more susceptible to TB. Female patients had lower levels of IL-17 and higher levels of IL-12 compared with male patients; however these findings do not support that female preponderance is due to differences in immune response.

## Figures and Tables

**Table 1:** The characteristics of the study population.

	Adults (*n* = 61)	Children (*n* = 25)
Age in years	Median 28, range 16–75	Median 9, range 2–14
Males (*n*)	27	10
Females (*n*)	34	15
Culture positive (*n*)	13/61^∗^	3/23^∗^
HIV positive (*n*)	5/44^∗^	0/12^∗^
BCG vaccinated (*n*)	45/57^∗^	21/25^∗^

*n* = number of cases; ^∗^denominator denotes the number of cases where results were available.

**Table 2:** Serum cytokine levels in the adult and paediatric patients with tuberculous lymphadenitis and healthy controls.

Cytokine (pg/mL)	Adult patients (*n* = 61) Median (range)	Healthy controls (*n* = 23) Median (range)	*P* value^∗^	Pediatric patients (*n* = 25) Median (range)	*P* value^∗∗^
IFN-*γ*	0.0 (0–42.8)	0.0 (0–1.5)	0.001	0.0 (0–40.4)	0.268
TNF-*α*	8.4 (0–352.5)	2.5 (0–51.7)	0.005	3.2 (0–33.4)	0.015
GM-CSF	35.2 (0–506.9)	0.0 (0–379.2)	0.007	0.0 (0–147.9)	0.023
IL-1*β*	48.7 (0–6305.4)	0.0 (0–933.2)	0.004	4.0 (0–610.5)	0.338
IL-2	0.0 (0–28.2)	0.3 (0–16.3)	0.038	1.0 (0–105.6)	0.003
IL-4	0.0 (0–18.5)	0.0 (0–4.3)	0.002	0.0 (0–5.4)	0.208
IL-5	1.0 (0–10.0)	0.5 (0–12.5)	0.559	0.0 (0–1.5)	0.001
IL-6	47.4 (0–25781.5)	0.0 (0–9355.1)	0.000	44.1 (0–587.4)	0.244
IL-8	370.4 (0–386578.0)	65.9 (1.6–2888.2)	0.071	68.2 (0–3551.4)	0.195
IL-10	0.0 (0–29.3)	0.0 (0–36.2)	0.560	0.0 (0–9.9)	0.233
IL-12	0.0 (0–37.8)	2.7 (0–15.3)	0.000	0.0 (0–9.5)	0.701
IL-15	0.0 (0–61.4)	0.0 (0–12.7)	0.812	0.6 (0–53.3)	0.385
IL-17	0.0 (0–133.7)	0	0.282	0	0.262

^∗^ *P* value for difference between adult patients and healthy controls and ^∗∗^ *P* value for difference between adult and pediatric patients.

**Table 3:** Relationship between the serum levels of cytokines in adult patients with tuberculous lymphadenitis (*n* = 61) based on Spearman's rank correlation. The values shown are the correlation coefficients.

	TNF-*α*	GM-CSF	IL-1*β*	IL-2	IL-4	IL-5	IL-6	IL-8	IL-10	IL-12	IL-15	IL-17
IFN-*γ*	.729^∗∗^	.741^∗∗^	.723^∗∗^	.058	.018	.252^∗^	.732^∗∗^	.551^∗∗^	.335^∗∗^	−.193	−.025	.178
TNF-*α*		.828^∗∗^	.724^∗∗^	.081	.055	.217	.674^∗∗^	.577^∗∗^	.315^∗^	−.035	.119	−.103
GM-CSF			.723^∗∗^	.062	−.012	.230	.794^∗∗^	.651^∗∗^	.210	−.063	.075	−.051
IL-1*β*				.213	.312^∗^	.287^∗^	.687^∗∗^	.539^∗∗^	.275^∗^	−.109	.208	.154
IL-2					.150	.275^∗^	.076	−.028	.181	.183	.454^∗∗^	.086
IL-4						.061	−.012	.068	.034	−.207	.037	.251
IL-5							.138	.261^∗^	.241	.022	.188	−.134
IL-6								.597^∗∗^	.066	−.038	−.050	.050
IL-8									.123	−.011	−.057	.076
IL-10										−.043	.068	.218
IL-12											.459^∗∗^	−.171
IL-15												.011

^∗∗^Correlation is significant at the 0.01 level (2-tailed). ^∗^Correlation is significant at the 0.05 level (2-tailed).

**Table 4:** Relationship between the serum levels of cytokines in children with tuberculous lymphadenitis (*n* = 25) based on Spearman's rank correlation. The values shown are the correlation coefficients.

	TNF-*α*	GM-CSF	IL-1*β*	IL-2	IL-4	IL-5	IL-6	IL-8	IL-10	IL-12	IL-15
IFN-*γ*	.566^∗∗^	.716^∗∗^	.369	−.244	.389	.274	.440^∗^	.497^∗^	.151	−.200	−.111
TNF-*α*		.802^∗∗^	.611^∗∗^	.028	.213	.303	.528^∗∗^	.648^∗∗^	.108	−.047	.291
GM-CSF			.726^∗∗^	.039	.442^∗^	.527^∗∗^	.758^∗∗^	.875^∗∗^	.026	−.191	.154
IL-1				.336	.490^∗^	.646^∗∗^	.596^∗∗^	.671^∗∗^	−.176	−.114	.424^∗^
IL-2					−.226	−.039	.122	.150	−.133	.256	.448^∗^
IL-4						.745^∗∗^	.341	.376	.008	−.391	−.076
IL-5							.401^∗^	.469^∗^	.070	−.538^∗∗^	−.038
IL-6								.842^∗∗^	−.190	−.282	.072
IL-8									−.068	−.115	.194
IL-10										−.259	−.474^∗^
IL-12											.530^∗∗^

^∗∗^Correlation is significant at the 0.01 level (2-tailed). ^∗^Correlation is significant at the 0.05 level (2-tailed).

**Table 5:** Differences in the serum cytokine levels in the adult Tanzanian patients based on gender and mycobacterial culture from lymph nodes.

Cytokine (pg/mL)	Male (*n* = 27) Median (range)	Female (*n* = 34) Median (range)	*P* value^∗^	Culture positive (*n* = 13) Median (range)	Culture negative (*n* = 48) Median (range)	*P* value^∗∗^
IFN-*γ*	0.0 (0–42.8)	0.0 (0–30.9)	0.98	0.0 (0–22.4)	0.0 (0–42.8)	0.383
TNF-*α*	3.2 (0–352.5)	11.8 (0–73)	0.15	8.4 (0.5–352.5)	12.4 (0–72.9)	0.874
GM-CSF	35.2 (0–507)	55.1 (0–457)	0.96	35.2 (0–506.9)	34.5 (0–457.7)	0.864
IL-1*β*	72.6 (0–1338)	5.7 (0–6305)	0.51	0.0 (0–1338.8)	66.5 (0–6305.4)	0.746
IL-2	0.0 (0–28)	0.0 (0–19.8)	0.31	0.0 (0–28.2)	0.0 (0–19.8)	0.193
IL-4	0 (0–9.5)	0 (0–18)	0.30	0.0 (0–6.8)	0.0 (0–18.5)	0.360
IL-5	1.5 (0–10)	0.75 (0–6.4)	0.16	1.0 (0–8.1)	1.0 (0–10.0)	0.465
IL-6	46.4 (0–18979)	55.5 (0–25781)	0.80	46.4 (0–18979.1)	55.6 (0–25781.5)	0.825
IL-8	362 (0–27670)	419 (0–386577)	0.70	638.2 (0–27670.3)	366.4 (0–386578.0)	0.486
IL-10	0.0 (0–29)	0.0 (0–22)	0.96	0.0 (0–29.3)	0.0 (0–22.8)	0.513
IL-12	0 (0–20.7)	0.1 (0–37)	0.03	0.3 (0–20.7)	0.0 (0–37.8)	0.029
IL-15	0.0 (0–61)	0.0 (0–25)	0.77	0.6 (0–21.1)	0.0 (0–61.4)	0.223
IL-17	0 (0–134)	0	0.04	0.0 (0–26.1)	0.0 (0–133.7)	0.638

^∗^ *P* value for difference between males and females and ^∗∗^ *P* value for difference between culture positive and culture negative TB patients.
